# Characterising the Inhibitory Actions of Ceramide upon Insulin Signaling in Different Skeletal Muscle Cell Models: A Mechanistic Insight

**DOI:** 10.1371/journal.pone.0101865

**Published:** 2014-07-24

**Authors:** Rana Mahfouz, Rhéa Khoury, Agnieszka Blachnio-Zabielska, Sophie Turban, Nicolas Loiseau, Christopher Lipina, Clare Stretton, Olivier Bourron, Pascal Ferré, Fabienne Foufelle, Harinder S. Hundal, Eric Hajduch

**Affiliations:** 1 INSERM, UMR-S 1138, Centre de Recherche des Cordeliers, Paris, France; 2 Université Pierre et Marie Curie – Paris 6, UMR-S 1138, Paris, France; 3 Université Paris Descartes, UMR-S 1138, Paris, France; 4 Department of Physiology, Medical University of Bialystok, Bialystok, Poland; 5 Division of Cell Signalling and Immunology, College of Life Sciences, University of Dundee, Dundee, United Kingdom; 6 INRA, UMR1331 Toxalim, Research Centre in Food Toxicology, Toulouse, France; 7 Département de Diabétologie et Maladies métaboliques, AP-HP, Hôpital Pitié-Salpêtrière, Paris, France; INSERM/UMR 1048, France

## Abstract

Ceramides are known to promote insulin resistance in a number of metabolically important tissues including skeletal muscle, the predominant site of insulin-stimulated glucose disposal. Depending on cell type, these lipid intermediates have been shown to inhibit protein kinase B (PKB/Akt), a key mediator of the metabolic actions of insulin, via two distinct pathways: one involving the action of atypical protein kinase C (aPKC) isoforms, and the second dependent on protein phosphatase-2A (PP2A). The main aim of this study was to explore the mechanisms by which ceramide inhibits PKB/Akt in three different skeletal muscle-derived cell culture models; rat L6 myotubes, mouse C2C12 myotubes and primary human skeletal muscle cells. Our findings indicate that the mechanism by which ceramide acts to repress PKB/Akt is related to the myocellular abundance of caveolin-enriched domains (CEM) present at the plasma membrane. Here, we show that ceramide-enriched-CEMs are markedly more abundant in L6 myotubes compared to C2C12 myotubes, consistent with their previously reported role in coordinating aPKC-directed repression of PKB/Akt in L6 muscle cells. In contrast, a PP2A-dependent pathway predominantly mediates ceramide-induced inhibition of PKB/Akt in C2C12 myotubes. In addition, we demonstrate for the first time that ceramide engages an aPKC-dependent pathway to suppress insulin-induced PKB/Akt activation in palmitate-treated cultured human muscle cells as well as in muscle cells from diabetic patients. Collectively, this work identifies key mechanistic differences, which may be linked to variations in plasma membrane composition, underlying the insulin-desensitising effects of ceramide in different skeletal muscle cell models that are extensively used in signal transduction and metabolic studies.

## Introduction

Once bound to its receptor, insulin stimulates a signalling network that functions to regulate whole-body glucose homeostasis by coordinating numerous physiological processes. Defects in the activation of insulin-induced signalling cascades are often associated with insulin resistance, a characteristic feature of obesity and type 2 diabetes [1]. The mechanisms by which insulin resistance develops are not yet fully understood but recent work has shown that forcing cells to accumulate fatty acids beyond their storage capacity may lead to insulin desensitisation through the generation of toxic lipid intermediates such as ceramide [2], [3].

Skeletal muscle is the major tissue responsible for insulin-stimulated glucose disposal and therefore considered as a primary target in the onset of insulin resistance. Various studies have suggested that ectopic accumulation of ceramide in response to oversupply of saturated fatty acids including palmitate may underlie the development of insulin resistance in this tissue [4]–[6].

Indeed, we and others have demonstrated that ceramide can impair insulin action through inhibition of protein kinase B (PKB/Akt), a key signal transduction intermediate that plays a pivotal role in coordinating the insulin-dependent uptake and utilization of glucose [7], [8]. Two skeletal muscle cell lines that have been extensively used to study the deleterious effects of ceramide upon insulin action are rat L6 and mouse C2C12 muscle cells. In differentiated rat L6 myotubes, treatment with palmitate or exogenous ceramide leads to the activation of the atypical protein kinase C isoform PKCζ which in turn directly interacts with and phosphorylates the pleckstrin homolog (PH) domain of PKB/Akt at Thr34. As a result of this interaction, PKB/Akt becomes sequestered into specialized domains of the plasma membrane known as caveolin-enriched microdomains (CEM) thereby preventing its recruitment to PIP3-enriched regions where it is normally activated in response to insulin [9]–[11]. In contrast, whilst exposure of C2C12 myotubes to palmitate has also been shown to result in the activation of aPKCζ, PKB/Akt becomes repressed primarily through its dephosphorylation by protein phosphatase 2A (PP2A) [12].

In this study, we set out to explore this differential mode of inhibition by ceramide upon insulin signalling in L6 and C2C12 myotubes. Importantly, we previously reported that in cells which lack CEM (e.g. fibroblasts), ceramide does not inhibit PKB/Akt through the “PKCζ-CEM” pathway, but rather through activation of PP2A [11]. This is in contrast to cells with abundant CEM (e.g. 3T3-L1 adipocytes) wherein ceramide acts to repress PKB/Akt via the PKCζ-CEM pathway [11]. We therefore hypothesised that variations in CEM content between these different muscle cell lines may account for the distinct signalling pathways utilised by ceramide to impair insulin action. Herein, we demonstrate that whilst ceramide impairs insulin-stimulated PKB/Akt activation *via* the ‘PKCζ-CEM’ pathway in L6 myotubes, this same lipid intermediate does so *via* a PP2A-dependent mechanism in C2C12 myotubes which exhibit a lower CEM content relative to L6 myotubes.

In addition, little is known about the functional mechanism(s) responsible for conveying the insulin desensitising effects of ceramide in human skeletal muscle. Therefore, we explored the potential involvement of the ‘PKCζ-CEM’ and PP2A pathways in primary cultured human muscle cells, which represent a good model for studying human skeletal muscle function [13] and can be modulated *ex vivo* [13]. We show that similar to L6 myotubes, it is the “PKCζ-CEM” pathway which predominantly mediates the repressive effects of ceramide upon PKB/Akt in human muscle cells.

## Methods

### Materials

All reagent-grade chemicals, insulin, palmitate, and BSA were purchased from Sigma-Aldrich. C_2_-ceramide was obtained from Cayman Chemical Company. Complete protein phosphatase inhibitor tablets were obtained from Boehringer-Roche Diagnostics. Antibodies against native PKB/Akt, Ser473-PKB/Akt and ^21/9^Ser GSK3α/β were from Cell Signalling (New England Biolabs), caveolin-3, PKCζ, PP2A and hemagglutinin from Santa Cruz Biotechnology and β-actin from Sigma-Aldrich. Horseradish peroxidase anti-rabbit, -mouse and -sheep/goat IgGs were from Jackson ImmunoResearch Laboratories and the enhanced chemiluminescent substrate was from Pierce-Perbio Biotechnology.

### Cell culture

L6 muscle cells were grown as a monolayer as described previously [14] to the stage of myotubes in α-minimum essential medium containing 2% fetal calf serum and 1% antimicotic/antibiotic solution. C2C12 myoblasts were maintained in Dulbecco’s modified Eagle’s medium (Gibco) containing 10%(v/v) fetal bovine serum. For differentiation into myotubes, the myoblasts were grown to confluence and the media were replaced with DMEM containing 2% horse serum. Both cell lines were maintained at 37°C in a humidified atmosphere of 5% CO_2_, 95% air. Myotubes were used for experiments 4 days following differentiation.

### Human skeletal muscle cells

Biopsies from healthy adult lean volunteers were obtained in the context of approved preclinical and clinical trials [15], and *via* the Tissue Bank for Research (Myobank) of the French Association against Myopathies (AFM), in agreement with the French bioethical law (law no 94–654 of 29 July 1994, modified 22 January 2002) on informed consent. Muscle samples from three adult type 2 diabetic patients were obtained from healthy tissue after leg amputation. Donors had no clinical signs of muscular disease and sampled tissues had no bacterial contamination. No clinical and biological infection has been observed (ultra-sensitive C reactive protein at 6.6+/−2.6 mg/L). At baseline, HbA1c and fasting glycaemia of type 2 diabetic patients were respectively 7.9+/−0.31% and 7.59+/−0.44 mmoles/L. Fresh muscle samples were minced and enzymatically dissociated with collagenase. Myoblasts were purified, grown and differentiated as myotubes as previously described [16].

### Preparation of whole cell lysates

Cells were lysed following experimental manipulation (see figure legends) in an appropriate volume of lysis buffer [14].

### Preparation of detergent-resistant membranes (DRM)

DRM were prepared as described previously [10]. Cells were homogenized into 25 mM MES (2-[N-Morpholino]ethanesulfonic acid), pH 6.0, 150 mM NaCl, 1% (w/v) Triton X-100, complete inhibitor tablet and lysate ran on a sucrose gradient. The gradient was centrifuged at 120,000 *g* for 20 h at 4°C. DRM fractions were then collected and frozen at −20°C until required.

### DRM-containing ceramide quantification

700 µL of each DRM fraction were freeze-dried and subsequently extracted once with chloroform/methanol (C/M) (2∶1), once with C/M (1∶1) and once with C/M (1∶2). The combined C/M extracts were freeze dried and solubilized in 2 mL of C/M (1∶1) with 3M of potassium hydroxide. The solution is incubated overnight at 50°C. 800 µL of distilled water (W) is added. The solution is neutralized using hydrochloric acid 1N. Organic extract was washed twice with C/M/W (3∶48∶47), dried under nitrogen and dissolved in 50 µL of C/M (1∶1) before analysis. High Performance Thin Layer Chromatography (HPTLC) analyses were performed with a multistep development. A first development was realized with the solvent system Hexane/diethyl ether/acetic acid (30∶15∶0.5) until a developing distance of 80 mm (from the lower edge of the plate). The second and third development was realized with the solvent system C/M/W (40∶10∶1) until the respective developing distance of 30 mm and 50 mm. The fourth development was realized with the solvent system C/M/acetic acid (47∶2∶0.5) until a developing distance of 80 mm. And the last development was realized with the solvent system Hexane/diethyl ether/acetic acid (30∶15∶0.5) until a developing distance of 80 mm. After each development the plate was dried 5 minutes. Bands were detected after immersion in a charring solution and drying at 250°C during 1 minute. Quantification was realized by densitometry with CAMAG TLC scanner 4 and winCATS software in absorption mode at 560 nm using a tungsten lamp.

### Analysis of sphingolipid content

Sphingolipids were extracted an assessed as described previously [17].

### Glucose transport

Human myotubes were washed rapidly with HEPES-buffered saline (HBS; 20 mM HEPES-Na (pH 7.4), 140 mM NaCl, 2.5 mM MgSO_4_, 5 mM KCl, 1 mM CaCl_2_), and glucose uptake was assayed by incubating cells with 10 µM 2-deoxy-[3H]-D-glucose (1 µCi/ml, 26.2 Ci/mmol) for 15 min in HBS. Carrier-mediated uptake was determined by quantitating cell-associated radioactivity in the presence of 10 mM cytochalasin B (an inhibitor of facilitative glucose transport). Radioactive medium was aspirated rapidly followed by three cell washes in ice-cold isotonic saline solution (0.9% NaCl, w/v) prior to lysis in 0.05 M NaOH [18]. Cell-associated radioactivity was determined by liquid scintillation counting and protein determined by the method of Bradford [19].

### Immunoblotting

Cell lysates were subjected to SDS/PAGE and immunoblotted as previously reported [14]. Nitrocellulose membranes were probed with various antibodies as described in the figure legends. Detection of primary antibodies were performed using appropriate peroxidase-conjugated IgGs and protein signals were visualized using enhanced chemiluminescence (Thermo Scientific Pierce) by exposure to Kodak autoradiographic film.

### Statistical analysis

Statistical analysis was carried out using a Student’s t test. Data were considered statistically significant at p values <0.05 and notified by *.

## Results

**Figure 1:**
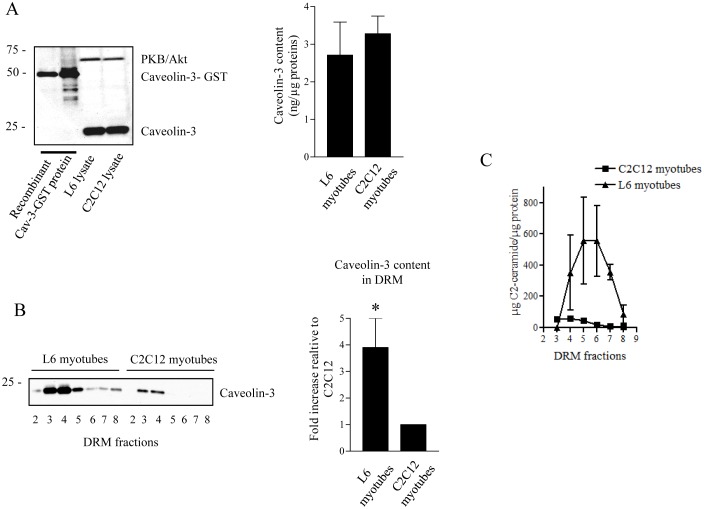
Levels of caveolin expression and ceramide content in both L6 and C2C12 myotubes. A. 15 µg of L6 and C2C12 lysates were immunoblotted alongside 65 ng and 130 ng of recombinant caveolin-3-GST (cav-3-GST) protein with an antibody against caveolin-3 (left panel). Bands were quantified and compared to known quantities of recombinant cav-3 proteins. Results were expressed as ng of cav-3 protein per µg of L6 or C2C12 protein lysat (right panel). B. L6 and C2C12 myotubes were solubilized in 1% Triton X-100 at 4°C and fractionated on sucrose gradients to isolate detergent resistant membranes (DRM) as described in the Methods section. Resulting fractions were collected from top to bottom of the gradient. Equal amounts of protein (1 µg) from fractions 2 to 8 of the sucrose gradient were then immunoblotted using an anti-caveolin-3 antibody. Bands were quantified and caveolin-3 content in L6 cells was expressed as fold increase relative to caveolin-3 content in C2C12 cells. * Significant change p<0.05 relative to C2C12 cells. These are representative of at least three independent experiments. C. Total ceramide content was quantified in DRM-containing fractions (3–8 of the sucrose gradient) from both L6 and C2C12 myotubes. These are representative of three independent experiments.

In an initial attempt to identify factors underpinning the distinct mechanisms involved in mediating the repressive effects of ceramide upon PKB/Akt in L6 and C2C12 myotubes through PKCζ and PP2A respectively, we hypothesised that differences in CEM abundance and/or composition may account for these differential modes of action. This was based on our previous published work demonstrating the key role that CEM abundance plays in determining ceramide-induced pathways leading to PKB/Akt inhibition in non-muscle cell types [11]. We selected caveolin-3 as a CEM marker since this protein is the predominant caveolin isoform expressed in muscle cells and is known to play a crucial role in maintaining CEM structure [20]. Immunoblot analysis of caveolin-3 revealed no significant difference in total caveolin-3 protein content in whole cell lysates of fully differentiated L6 and C2C12 muscle cells (around 3 ng caveolin-3 per µg total protein lysate), as shown in figure 1A. We next examined caveolin-3 protein abundance in CEM structures by isolating detergent resistant membranes (DRM) on discontinuous sucrose density gradients [10], and immunoblotting the resulting DRM fractions for caveolin-3. Interestingly, Figure 1B shows that caveolin-3 protein expression in DRM (fractions 3 to 5) obtained from L6 myotubes was approximately 3-fold greater compared to those isolated from C2C12 myotubes. This suggests higher CEM abundance in L6 versus C2C12 plasma membranes. Because ceramide is known to accumulate within CEMs [21], we assessed whether ceramide concentration would correlate with CEM caveolin-3 content in DRM fractions isolated from these two muscle cell lines. To investigate this, we treated L6 and C2C12 myotubes with 100 µM short-chain C2-ceramide for two hours prior to isolation of DRM fractions that were subsequently used for quantitative determination of ceramide content. Figure 1 C shows that ceramide accumulates in DRM fractions 3 to 6 of both cell lines but to a far lesser extent (10 times less in terms of abundance) in C2C12 compared to L6 myotubes. Therefore, lower CEM abundance in C2C12 myotubes may account for the reduced capacity of these cells to retain ceramide in active membrane microdomains. This may also help to explain why inhibition of PKB/Akt by ceramide occurs outside these membrane domains and is mainly dependent upon PP2A activation in C2C12 myotubes [8]–[10].

**Figure 2:**
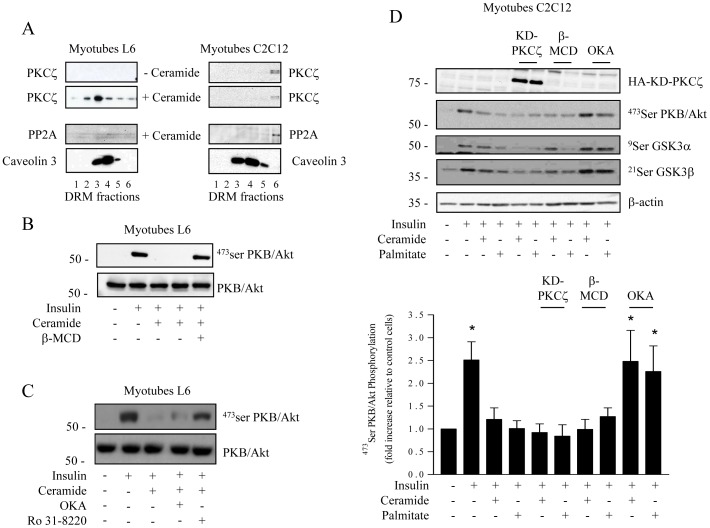
Mechanism of ceramide action on the insulin signalling pathway in both L6 and C2C12 myotubes. A. L6 and C2C12 myotubes were treated with 100 µM C2-ceramide for 2 h before isolation of DRMs. Equal amounts of protein (1 µg) of each fraction were then immunoblotted for the presence of PKCζ, PP2A and caveolin-3. These are representative immunoblots from three independent experiments. B. L6 myotubes were pre-incubated with 100 µM C2-ceramide for 2 h and with 5 mM MβCD for the last 30 min. Cells were then stimulated with 100 nM insulin for 10 min before being lysed and immunoblotted using either a phospho-specific antibody directed against Ser^473^PKB/Akt or a pan PKB antibody. C. Control C2C12 myotubes or KD-PKCζ-infected C2C12 myotubes were incubated with 100 µM C2-ceramide for 2 h or with 0.75 mM palmitate for 16 h. Control C2C12 myotubes were then treated with 500 nM okadaic acid (OKA) or with 5 mM MβCD the last 30 min. All cells were treated with 100 nM insulin for the last 10 min before being lysed. Cell lysates were immunoblotted with antibodies against native PKB/Akt, Ser^473^PKB/Akt, Ser^21/9^GSK3 α/β, β-actin and hemagglutinin (HA). Scanning densitometry was performed to quantify changes in Ser^473^PKB/Akt abundance in cell lysates. Bars represent mean +/− SEM. * Significant change p<0.05 relative to the untreated control. Blots shown represent at least three separate experiments.

To test this possibility, we examined whether the “PKCζ-CEM” pathway which inhibits PKB/Akt in L6 myotubes may also be functional in C2C12 myotubes. As predicted based on comparative CEM analysis, whilst C2-ceramide promotes the recruitment of PKCζ into L6 CEM domains, no PKCζ was detected in CEMs (fractions 3–5) isolated from C2C12 myotubes challenged with C2-ceramide (figure 2A). Furthermore, PP2A was not detected in ceramide-treated CEM from either muscle cell lines (figure 2A) in accordance with previous work showing that ceramide-activated PP2A acts outside the CEM environment [11].

Having established that PKCζ is not recruited to CEM in response to ceramide in C2C12 myotubes, we next assessed whether the presence of CEM was dispensable for ceramide to repress PKB/Akt in these cells. Figure 2B shows that in L6 myotubes, ceramide-mediated inhibition of PKB/AktSer473 phosphorylation in response to insulin was prevented by methyl β-cyclodextrin (MβCD), a cholesterol depleting agent that has been shown to disrupt CEM integrity without affecting insulin signalling capacity [10]. This demonstrates the importance of CEM in supporting ceramide action in these cells. Moreover, we used either okadaic acid (OKA), a potent inhibitor of protein phosphatase 2A (PP2A), and the pharmacological Protein Kinase C (PKC) inhibitor Ro 31.8220 to determine which ceramide-activated pathway (aPKCζ or PP2A) mediates the repressive effect of ceramide on PKB/Akt. The bisindolemaleimide Ro 31.8220 potently inhibits conventional and novel PKCs in an ATP-competitive manner with IC50 values in the submicromolar range, as well as atypical PKCs in the micromolar range [9]. Figure 2C shows that whilst applying OKA (500 nM) displays no significant effect, Ro 31.8220 (at 5 µM, optimal final concentration to counteract the activation of aPKCζ by ceramide in L6 myotubes, figure S1A) is able to largely prevent the negative action of ceramide upon the insulin-induced phosphorylation of PKB/Akt, thereby confirming the importance of the “CEM- PKCζ” pathway in mediating this insulin desensitising effect in L6 myotubes.

In contrast, in C2C12 myotubes, treatment with MβCD did not prevent the repressive action of either palmitate (ceramide precursor) or C2-ceramide upon insulin-stimulated PKB/Akt Ser473 phosphorylation, or upon phosphorylation of one of its physiological downstream targets, GSK3α/β (figure 2D). The lack of CEM involvement in ceramide action in C2C12 myotubes was further confirmed by overexpressing an inactive dominant negative PKCζ mutant (KD- PKCζ) in these cells. Figure 2D shows that, in contrast to what has previously been reported in L6 myotubes [9], prevention of PKCζ activation does not counteract either palmitate nor ceramide mediated repression of insulin-induced PKB/Akt and GSK3α/β phosphorylation. Furthermore, inhibition of PP2A using OKA (at 500nM, optimal final concentration to counteract the activation of PP2A by ceramide in C2C12 myotubes, figure S1B) was found to completely prevent palmitate and ceramide mediated suppression of PKB/Akt and GSK3α/β phosphorylation in insulin treated C2C12 myotubes (figure 2D). Together, these observations suggest that, depending on cellular CEM abundance, fatty acid overload can inhibit insulin induced PKB signalling through at least one of two distinct mechanisms.

We next explored which of these mechanisms is likely to predominate in human muscle cells.

**Figure 3:**
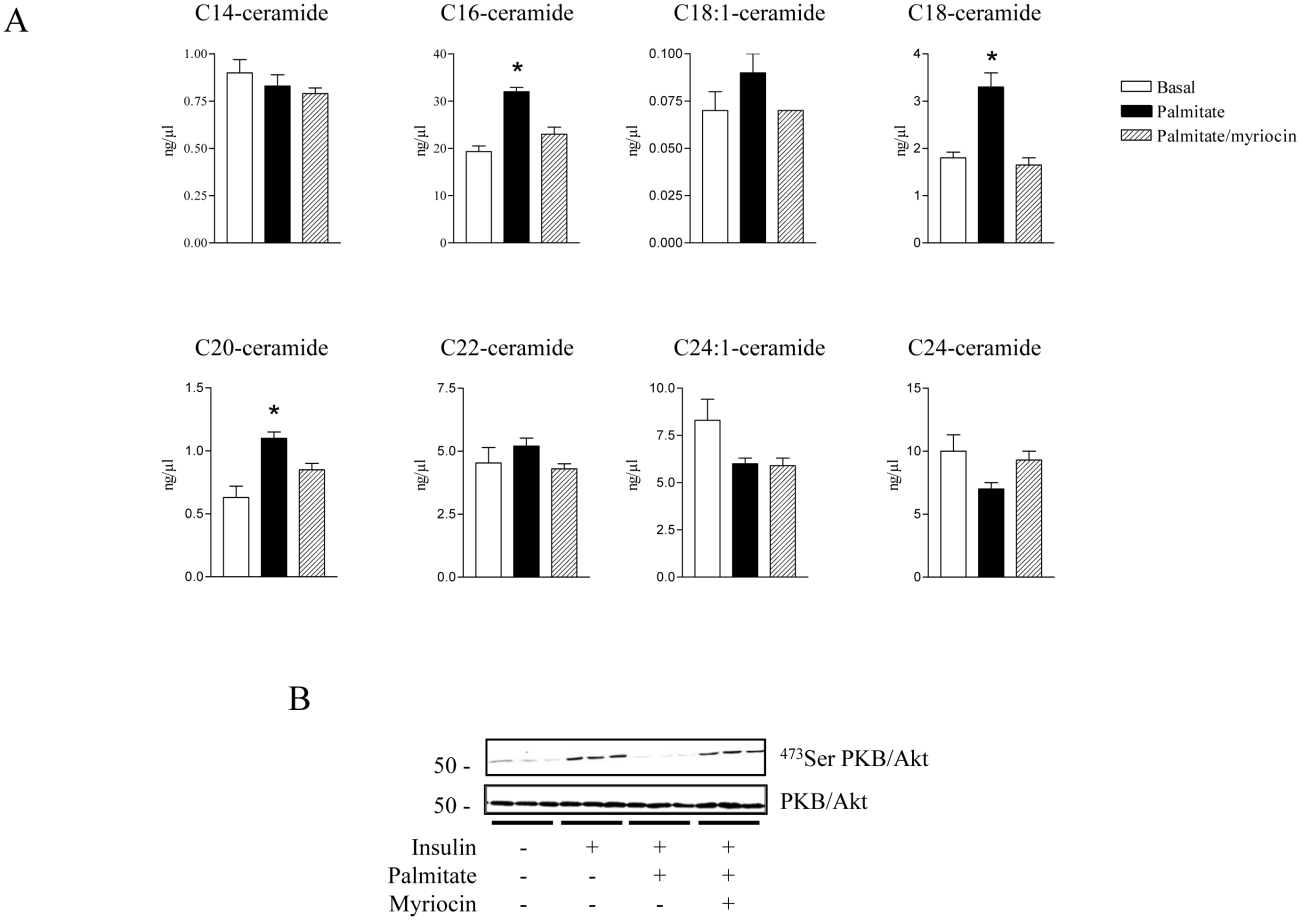
Effect of Palmitate on ceramide production and insulin sensitivity in human muscle cells. Human myotubes were incubated with 0.75 µM myriocin. A. Following this incubation, muscle cells were harvested in ice-cold PBS and lipids extracted to assess ceramide species content as described in the Methods section. Bars represent mean ± SEM from 3 separate experiments and * denote a significant difference from the untreated control values (P<0.05). B. Cells were then stimulated with 100 nM insulin for the last 10 min before being lysed and immunoblotted using either a phospho-specific antibody directed against Ser^473^PKB/Akt or a pan PKB antibody.

To investigate this, we used primary cultures of muscle cells derived from human biopsies. These cells have been shown to differentiate into myotubes in culture and are insulin responsive [16]. First, we wanted to establish whether our primary human muscle cells responded to palmitate treatment through assessing their ability to generate ceramide. Figure 3A shows that incubation of cultured human myotubes with 0.75 mM palmitate for 24 h led to significant increases in the cellular content of three distinct ceramide species (i.e. C16-, C18-, and C20 ceramides). Importantly, the ability of palmitate to promote these elevations in intracellular ceramide was attenuated in the presence of myriocin, a pharmacological inhibitor of the enzyme serine palmitoyl transferase (SPT) which catalyses the initial rate limiting step of *de novo* ceramide synthesis [22].

Subsequently, we assessed whether endogenous ceramide production from palmitate would impair insulin action in human muscle cells. Figure 3B shows that prolonged (24 h) incubation of human myotubes with palmitate completely abrogated insulin-induced PKB/Akt Ser473 phosphorylation. However, when human myotubes were pre-incubated with myriocin, the insulin desensitising effect of palmitate was completely prevented, thereby highlighting the important role that ceramide accumulation plays in mediating palmitate induced insulin resistance in human muscle cells (figure 3B).

**Figure 4:**
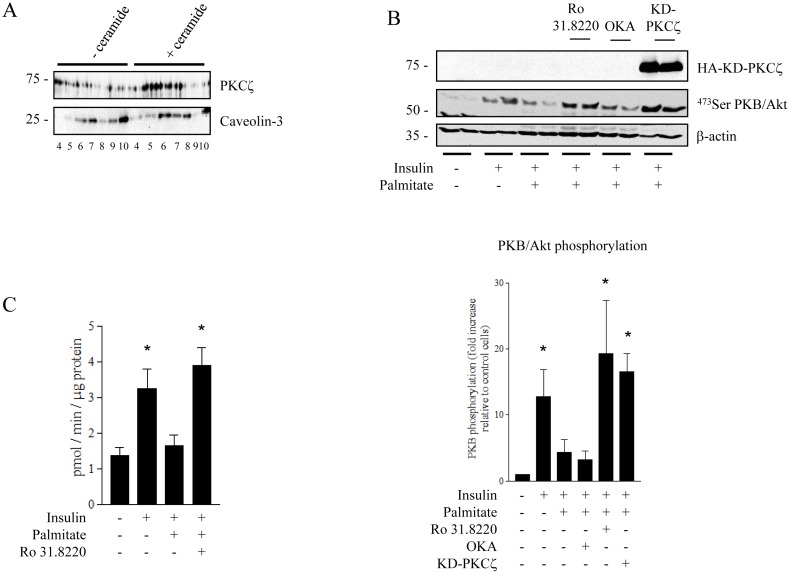
Ceramide action on the insulin signalling pathway in human myotubes. A. Human myotubes were treated with 100 µM C2-ceramide for 2 h before isolation of DRMs. Equal amounts of protein (1 µg) of each fraction were then immunoblotted for the presence of PKCζ and caveolin-3. These are representative immunoblots from three independent experiments. B. Human myotubes from control donors were treated with 0.75 mM palmitate for 48 h in the presence or absence of Ro 31.8220 (5 µM, 48 h) or OKA (500 nM, last 30 min). KD-PKCζ-infected human myotubes were treated with 0.75 mM palmitate for 48 h. Cells were then stimulated with 100 nM insulin for the last 10 min before being lysed. Cell lysates were immunoblotted with antibodies against Ser^473^PKB/Akt, β-actin and hemagglutinin (HA). Scanning densitometry was performed to quantify changes in Ser^473^PKB/Akt abundance in cell lysates. Bars represent mean +/− SEM. * denotes significant change p<0.05 relative to the untreated control. Blots shown represent at least three separate experiments. C. Human myotubes were incubated with 0.75 mM palmitate for 48 h in the presence or absence of Ro 31.8220. Cells were then treated with 100 nM insulin for the last 30 min prior to the measurement of 2-deoxy-glucose uptake as described in the experimental section. Bars represent mean +/− SEM. * Significant change p<0.05 relative to the untreated control (n = 3).

Next, we explored the mechanism by which ceramide inhibits insulin signalling in human muscle cells. To do this, we treated human myotubes with C2-ceramide for 2 h prior isolating CEM domains. As observed in L6 myotubes (figure 2A), C2-ceramide treatment induced recruitment of PKCζ into CEM domains of human myotubes (figure 4A). Then, to determine whether the ceramide-activated PKCζ pathway was involved in repressing PKB/Akt activity in human muscle cells, we suppressed ceramide induced activation of PKCζ using either Ro 31.8220 (5 µM), or by overexpressing the inactive kinase-dead (KD)-PKCζ mutant in these cells. Figure 4B shows that application of Ro 31.8220 or expression of the KD- PKCζ mutant were able to prevent the ability of palmitate to reduce insulin-stimulated phosphorylation of PKB/Akt in human cells. In contrast, the PP2A inhibitor okadaic acid did not counteract the repressive action of palmitate towards PKB/Akt (figure 4B). In accordance with its ability to induce PKB/Akt activation, insulin stimulated glucose uptake by over 2-fold in control human myotubes (figure 4C). Importantly, whilst palmitate provision led to a profound reduction in insulin-induced glucose transport, this inhibition was completely prevented in human muscle cells cotreated with Ro 31.8220 (figure 4C). These findings indicate that, in human muscle cells, ceramide acts to impair PKB/Akt predominantly through activation of the PKCζ pathway.

**Figure 5:**
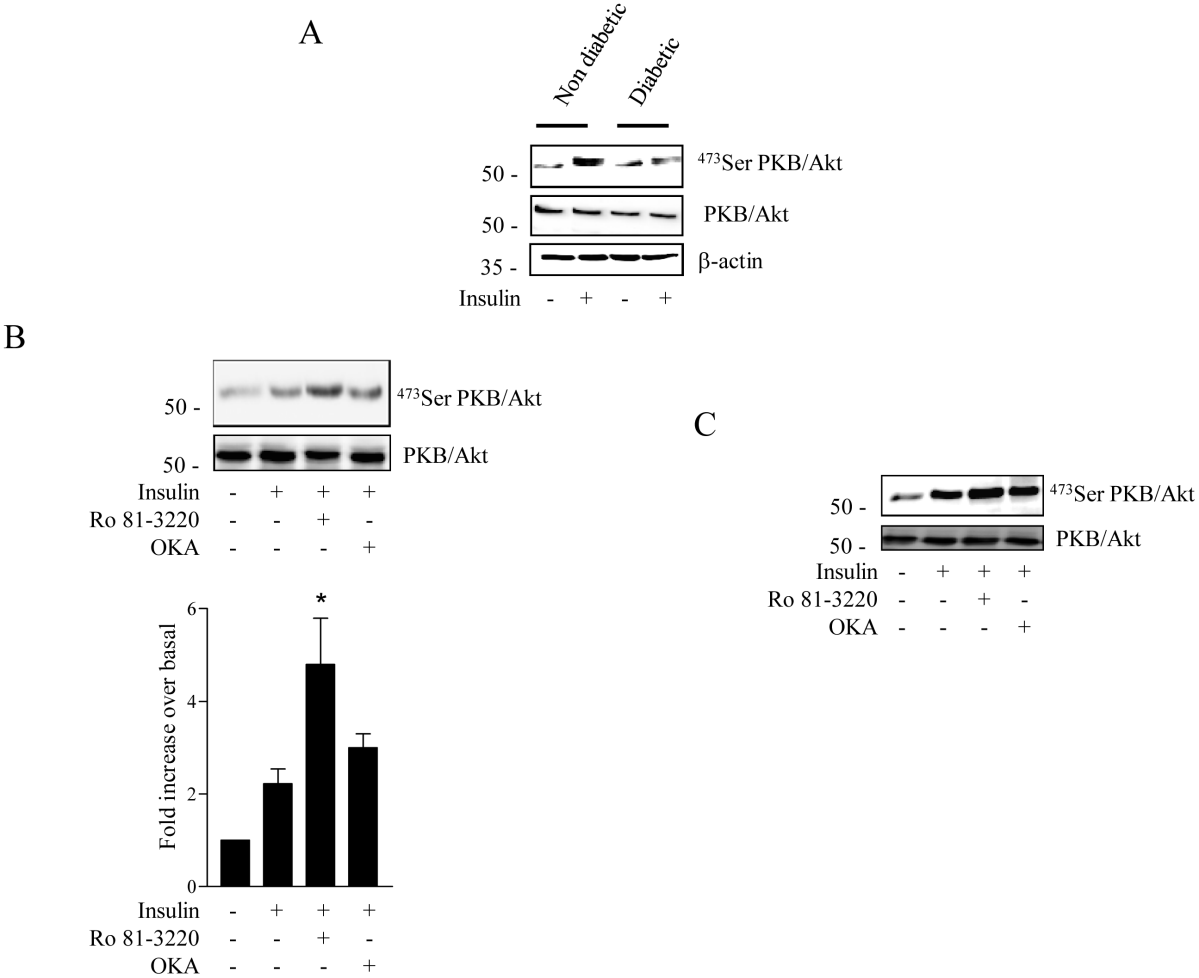
Inhibition of aPKCs sensitizes insulin-resistant human myotubes to insulin. A. Differentiated myotubes from control donors were incubated with Ro 31.8220 (5 µM, 18 h) or OKA (500 nM, last 30 min), then stimulated with 100 nM insulin for the last 10 min before being lysed. Cell lysates were immunoblotted with antibodies against Ser^473^PKB/Akt, total PKB/Akt and β-actin. B. Differentiated myotubes from diabetic patients were incubated with Ro 31.8220 (5 µM, 18 h) or OKA (500 nM, last 30 min), then stimulated with 100 nM insulin for the last 10 min before being lysed. Cell lysates were immunoblotted with antibodies against Ser^473^PKB/Akt and total PKB/Akt. Scanning densitometry was performed to quantify changes in Ser^473^PKB/Akt abundance over total PKB/Akt protein expression in cell lysates. Bars represent mean +/− SEM. * denotes significant change p<0.05 relative to the insulin treated cells. Blots represent three separate experiments.

Several studies have previously demonstrated that myotubes derived from type 2 diabetic patients conserve important characteristics of the diabetic phenotype [23]–[25]. For example, insulin stimulated PKB/Akt phosphorylation in cultured human diabetic myotubes is reduced compared to that observed in non-diabetic human myotubes (figure 5A). Interestingly, pre-incubation with Ro 31.8220, but not OKA, markedly improved insulin responsiveness in diabetic myotubes (figure 5B). In contrast, neither Ro 31.8220 nor OKA significantly altered insulin stimulated PKB/Akt phosphorylation in human control myotubes (figure 5C).

Collectively these observations indicate that it is the PKCζ pathway which prevails in conveying the inhibition of PKB/Akt signalling by excess of lipids in the context of type 2 diabetes.

## Discussion

The findings presented in the current study indicate that the inhibitory effects of ceramide on insulin-induced PKB/Akt signalling can be mediated through two distinct mechanisms depending on muscle cell type. In rat L6 myotubes, ceramide acts by promoting the activation of PKCζ and its subsequent recruitment to plasma membrane CEMs. In contrast, in mouse C2C12 myotubes, ceramide represses PKB/Akt activity through stimulation of cytoplasmic phosphatase PP2A. Importantly, we show that differences in CEM abundance may be an important factor in determining which mechanism is prominent within any particular cell type.

One possible explanation for the observed differences in CEM abundance and distinct modes of ceramide action in these two muscle cell lines, may be partly due to their contrasting abilities to differentiate from myoblasts into mature myotubes. Both L6 and C2C12 myoblasts exhibit characteristic features of muscle myogenesis, including migration and proliferation in medium containing 10% (v/v) foetal calf serum, followed by elongation and fusion to form myotubes following serum reduction. Interestingly, previous studies have shown that L6 cells differentiate readily into myotubes when maintained in medium containing 2% (v/v) serum, whereas C2C12 differentiation is more pronounced in serum-free medium compared to a serum-containing system [26], [27]. Thus, under standard conditions used to differentiate both cell lines (i.e. 2% (w/w) serum), it is possible that L6 myotubes mature more rapidly and/or more efficiently than C2C12 myotubes. However, since caveolins are late differentiation markers [28] and we see similar levels of caveolin-3 content in both differentiated cell types (figure 1A), this is unlikely to account for any mechanistic differences, and rather suggest that these two muscle cell models display different membrane characteristics, reinforcing the importance of our study.

Another key finding from this work concerns the identification of the process by which ceramide mediates insulin desensitisation in human muscle cells. To investigate this, we utilised human muscle satellite cells obtained from either insulin sensitive or diabetic donors that we differentiated *in vitro* into mature myotubes. There are several advantages using this cell model, including its morphological, biochemical and metabolic similarity to adult skeletal muscle cells [13]. Importantly, these cultured myotubes have been shown to maintain metabolic properties of the donor [13]. Similar to what we observed in L6 and C2C12 myotubes, we found that treatment of cultured insulin sensitive human myotubes with palmitate led to both an increase in the biosynthesis of three ceramide species (C16-, C18- and C20-ceramides) and an associated reduction in insulin stimulated PKB/Akt activation. Interestingly, the levels of similar ceramide species (C16- and C18-ceramides) have also previously been reported to be increased in myotubes obtained from type 2 diabetic individuals compared to controls [6]. Therefore, these observations suggest that these specific ceramide species may play a key role in mediating the deleterious effects of palmitate upon insulin signal transduction. In accordance with this, we demonstrate that inhibition of endogenous ceramide production with myriocin abolishes palmitate-induced insulin resistance in our human myotubes. Furthermore, in line with our observations in L6 muscle cells [8], [9], palmitate-derived ceramide acts to inhibit insulin signalling through activation of the “PKCζ-CEM” pathway in human myotubes. Importantly, we confirmed this by using human myotubes that have been made insulin resistant through palmitate exposure, as well as using primary culture of skeletal muscle cells from diabetic patients. It is important to note that the latter cell model has been extensively studied and displays several metabolic defects that characterize *in vivo* insulin resistance of skeletal muscle such as inhibition of insulin stimulated glucose uptake and glycogen synthesis [24], [25], [29], [30], concomitant with impaired insulin signalling [23], [31].

Together, these data implicate the “PKCζ-CEM” pathway as an important therapeutic target to counteract the deleterious effects of saturated fatty acids and their derived lipid intermediates upon insulin signalling in muscle cells. It is important to emphasise that myotubes derived from human subjects used in the present study could present altered membrane structure, gene expression and function due to *in vitro* culture and differentiation. Therefore, it would be interesting to confirm our results in intact muscle.

In summary, our study highlights key mechanistic differences by which ceramide can impair insulin action in rat L6 and mouse C2C12 myotubes. Importantly, our findings suggest that the effectiveness of distinct ceramide-induced pathways to promote insulin resistance in these cell lines, namely through activation of atypical PKC isoforms and PP2A respectively, may be determined by cellular CEM abundance and/or composition. Furthermore, we demonstrate for the first time that ceramide acts to repress insulin-induced PKB/Akt signalling in primary human myotubes through a pathway dependent on PKCζ. Together, these data raise awareness of the key role that cell membrane structure and/or composition may play in determining the effectiveness and/or predominance of those pathways which can potentially cause insulin resistance in different skeletal muscle cell models. Furthermore, our findings emphasise the need for caution when interpreting and comparing data from different muscle cell lines, particularly when exploring the role of fatty acid derived lipids such as ceramide in the modulation of insulin-regulated signalling and its associated processes.

## Supporting Information

Figure S1 **A: L6 myotubes treated with or without 100 µM C2-ceramide in the presence of different concentrations of Ro 31–8220 for 2 h, prior to stimulation with insulin (100 nM for 10 min).** B: C2C12 myotubes treated with or without 100 µM C2-ceramide for 2 h in the presence of different concentrations of OKA for the last 30 min, prior to stimulation with insulin (100 nM for 10 min).
(TIF)
