# Supplementary figures and images for: Characterising the Inhibitory Actions of Ceramide upon Insulin Signaling in Different Skeletal Muscle Cell Models: A Mechanistic Insight
Source: PLoS One. 2014 Jul 24;9(7):e101865. doi: 10.1371/journal.pone.0101865 (PMC4109934; doi:10.1371/journal.pone.0101865)

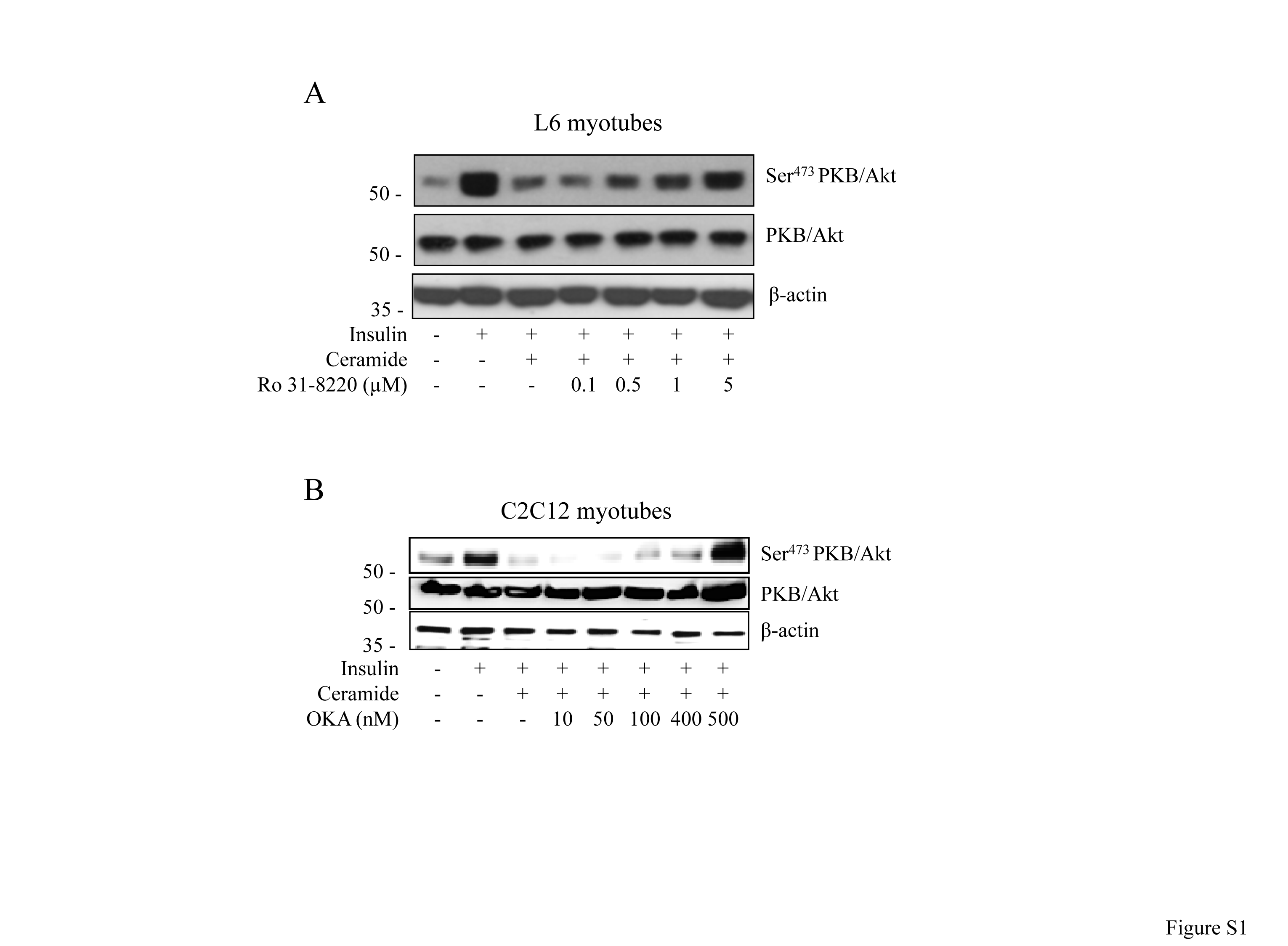

Supplement: Figure S1 — A: L6 myotubes treated with or without 100 µM C2-ceramide in the presence of different concentrations of Ro 31–8220 for 2 h, prior to stimulation with insulin (100 nM for 10 min). B: C2C12 myotubes treated with or without 100 µM C2-ceramide for 2 h in the presence of different concentrations of OKA for the last 30 min, prior to stimulation with insulin (100 nM for 10 min). (TIF) [file pone.0101865.s001.tif]
